# Postpartum hemorrhage incidence and risk factors: Evidence from a multicenter study in Zhejiang Province, China

**DOI:** 10.1371/journal.pone.0323190

**Published:** 2025-05-29

**Authors:** Xiaoyi Ding, Mustafe Abdi, Bingqing Liu, Yuanying Ma

**Affiliations:** 1 Department of Obstetrics and Gynecology, Xianlin Street Community Health Service Center, Hangzhou, China; 2 Department of Women’s Health, Women’s Hospital School of Medicine Zhejiang University, Hangzhou, China; American Hospital of Paris: Hopital Americain de Paris, FRANCE

## Abstract

**Objective:**

Postpartum hemorrhage (PPH) is a leading cause of maternal mortality worldwide, and its risk factors vary regionally. This study assessed the incidence and determinants of PPH in Zhejiang Province, China.

**Methods:**

We conducted a cross-sectional study using data from the Maternity Near Miss Surveillance System in Zhejiang Province, collected from January to December 2020. The cohort included 56,014 pregnant women at a gestational age of 28 weeks or more. PPH was defined as blood loss of ≥500 mL for vaginal deliveries and ≥1,000 mL for cesarean sections within 24 h of delivery. Logistic regression analyzed the risk factors for PPH.

**Results:**

Of the pregnant women, 2,016 (3.60%) experienced PPH. The mortality rate associated with PPH was 1.74 per 100,000 live births. Significant independent risk factors included multiple births (OR 3.10; 95% CI 2.42–3.98; *P* < 0.01), vaginal delivery (OR 3.00; 95% CI 2.62–3.42; *P* < 0.01), macrosomia (OR 2.30; 95% CI 1.97–2.68; *P* < 0.01), hypertensive disorders of pregnancy (OR 1.79; 95% CI 1.50–2.13; *P* < 0.01), and a lower educational level (OR 1.20; 95% CI 1.07–1.36; *P* < 0.01).

**Conclusion:**

The incidence and mortality rates of PPH in Zhejiang Province are low. Identified risk factors such as multiple births, vaginal delivery, macrosomia, hypertensive disorders, and lower education level can guide interventions to mitigate PPH risk.

## Introduction

Postpartum hemorrhage (PPH), defined as a blood loss exceeding 500 mL within 24 h following vaginal delivery or 1,000 mL post-cesarean section, is a major contributor to maternal mortality worldwide, accounting for over 10% of maternal deaths [1]. Although the direct maternal mortality from PPH in China is declining, its negative impacts on maternal and infant health remain significant. PPH is associated with complications such as maternal anemia, infections, hemorrhagic shock, and coagulopathy. While PPH does not directly correlate with perinatal outcomes, high-risk conditions can lead to adverse effects [2,3]. With the loosening of birth control policies in China, the incidence of PPH is increasing [4]. Therefore, early identification and management of risk factors are crucial for reducing maternal deaths and enhancing perinatal outcomes.

Studies on risk factors for PPH have varied widely, often limited by the scope of single-center studies. A case–control study of 462 women in France identified prior PPH, pre-eclampsia, and macrosomia as risk enhancers [5]. In Ethiopia, a study involving 1,060 women highlighted older maternal age (≥35 years), prolonged labor (>24 h), vaginal or cervical lacerations from instrumental births, and delivery management by medical interns as significant risks [6]. Other recognized risk factors across various studies include anemia, multiparity, obesity, placenta previa/accreta, coagulation disorders, and chorioamnionitis [7–11].

Reliable evidence regarding risk factors for PPH in the Chinese population is limited. Prior small, single-center studies have identified multiparity, maternal age ≥ 35 years, multiple pregnancies, cesarean delivery, a history of abortion, and macrosomia as risk factors. Since 2016, China has enforced a universal two-child policy in response to an aging population and declining birth rates. This policy change has significantly altered the demographic profile of Chinese pregnant women, who now tend to be older, possess lower educational levels, and exhibit higher rates of assisted reproductive technology use and complicated pregnancies than in the pre-policy era [12,13]. The applicability of previously identified risk factors under the new demographic conditions remains uncertain. Several single-center studies have investigated risk factors for PPH under this new policy but were limited by selection bias. Consequently, comprehensive, large-scale multicenter studies are essential to thoroughly explore risk factors for PPH in the current Chinese context.

This study employed data from the Zhejiang Province Maternal Near-Miss Surveillance System (MNMSS), which encompasses 18 surveillance institutions. The aim was to delineate the incidence of PPH and identify its risk factors, thereby providing a scientific foundation for reducing its incidence.

## Materials and methods

### Study design and population

This cross-sectional study utilized data from the Zhejiang Province MNMSS, established in October 2010 [14]. The system includes medical institutions with over 1,000 annual deliveries and is stratified by geographic and urban/rural characteristics to ensure representativeness. Nationwide, 326 institutions were sampled. The study population consisted of pregnant women admitted to obstetric departments or combined obstetrics and gynecology departments within these institutions. Women admitted to other departments or those receiving only miscarriage prevention treatments were excluded. In Zhejiang Province, the MNMSS covers 18 institutions (11 tertiary and seven secondary hospitals), comprising 12 county hospitals, four municipal hospitals, and two provincial hospitals. The details of the Zhejiang Province MNMSS and the data collection process are also described elsewhere [15]. Quality control is conducted by county, municipal, and provincial maternal and child health hospitals, which review all monitoring hospitals one to two times annually against jurisdictional standards. If a hospital’s error in reporting obstetric complications exceeds 5%, a complete data recheck is mandated. All institutions in Zhejiang Province have met these quality control standards.

The study included data from women who delivered at these 18 institutions between January and December 2020. Inclusion criteria were delivery at ≥28 weeks of gestation, hemorrhage within 24 h post-delivery, and a bleeding volume of ≥500 mL for vaginal delivery and ≥1,000 mL for cesarean section. Exclusion criteria included unreported or unclear hemorrhage volumes and the patient’s inability to cooperate with medical staff in completing survey forms. Of the 69,134 cases monitored, 56,960 met the inclusion criteria; 56,014 women were ultimately included in the study after excluding 946 due to unclear bleeding information (901) or non-cooperation (45). As this study relied on MNMSS data, it did not require informed patient consent. During data analysis, all information was anonymized to ensure that no personally identifiable information was included.

### Data collection and definition

Doctors and nurses at the 18 surveillance sites received training to prospectively collect data from admission to discharge using a tailored data collection form for each woman; this data was subsequently entered into a web-based management system. The forms gathered information on maternal demographics, pregnancy comorbidities, PPH, and perinatal and maternal outcomes. Maternal demographics encompassed maternal age, education level, number of prenatal checkups, parity, cesarean section history, delivery mode, and number of fetuses. Pregnancy comorbidities included hypertensive disorders (gestational hypertension; pre-eclampsia; eclampsia; HELLP syndrome), pregnancy-associated cardiac and liver diseases, gestational diabetes, and macrosomia. Perinatal and maternal outcomes covered maternal death, critical illness, neonatal birth status (live birth or stillbirth), discharge status, and Apgar scores. Details on the data collection process have been reported elsewhere [14]. Macrosomia was defined as a birth weight of ≥4,000 g, and PPH as blood loss of ≥500 mL for vaginal delivery and ≥1,000 mL for cesarean sections within 24 h post-delivery [16]. The consistency of PPH assessment and diagnosis was ensured across monitoring agencies, aligning with clinical practice norms.

### Statistical analysis

Statistical analysis was conducted using IBM SPSS 26.0. Normally distributed data were presented as means ± standard deviations (SD) and analyzed with the *t-test* for two-group comparisons. Non-normally distributed data were described using medians and interquartile ranges, with the Mann–Whitney *U* test employed for two-group comparisons. Categorical variables were expressed as *n* (%) and analyzed using the χ^2^ test or Fisher’s exact test. Logistic regression was applied to explore associations between risk factors and PPH, beginning with univariate logistic regression to assess baseline variables and subsequent adjustment for potential confounders. A multivariate logistic regression model was then constructed, positioning PPH as the dependent variable and including candidate factors as covariates, entered using the entry method. Differences were deemed statistically significant for two-sided *P*-values < 0.05.

### Ethics approval

Ethical approval for the Zhejiang Province MNMSS was granted by the ethics committee of West China Second University Hospital (protocol ID, 2012008). As this study utilized data from the Zhejiang Province MNMSS database without direct patient interaction or personal information collection, the requirement for informed consent was waived by the ethics committee.

## Results

The study included 56,014 pregnant women, with PPH diagnosed in 2,016 (3.60%). The average maternal age at delivery was 31.3 ± 4.7 years. 17.88% of the participants had a junior high school education or lower. Delivery modes included 32,460 vaginal (57.95%) and 23,554 cesarean (42.05%) deliveries. There were 1,396 (2.49%) cases of multiple births, 3,185 (5.69%) cases of macrosomia, and 2,997 (5.35%) cases of hypertensive disorders of pregnancy. There were two maternal deaths (3.50 per 100,000 live births), 248 (4.34‰) stillbirths, and 1,407 (2.46%) cases of neonatal asphyxia (Table 1).

**Table 1:** Comparison of the characteristics of the PPH and non-PPH groups.

Characteristics	Non-PPH *n* (%)/x̄ ± s	PPH *n* (%)/x̄ ± s	*P*
Age (years)			
<35	41,629 (77.09)	1,572 (77.98)	0.35
≥35	12,369 (22.91)	444 (22.02)	
Education level			
College or above	34,309 (63.54)	1,218 (60.42)	0.02
High school	10,067 (18.64)	406 (20.14)	
Junior high school or below	9,622 (17.82)	392 (19.44)	
Number of prenatal checkups			
<5	1,076 (1.99)	59 (2.93)	<0.01
≥5	52,922 (98.01)	1,957 (97.07)	
Parity			
Primipara	27,970 (51.79)	1,077 (53.42)	0.15
Multipara	26,028 (48.21)	939 (46.58)	
Mode of delivery			
Cesarean section	23,138 (42.85)	416 (20.63)	<0.01
Vaginal delivery	30,860 (57.15)	1,600 (79.37)	
History of cesarean section			
No	43,457 (80.48)	1,876 (93.06)	<0.01
Yes	10,541 (19.52)	140 (6.94)	
Hypertensive disorders of pregnancy			
No	51,153 (94.73)	1,864 (92.46)	<0.01
Yes	2,845 (5.27)	152 (7.54)	
Cardiac diseases			
No	53,755 (99.55)	2,003 (99.36)	0.20
Yes	243 (0.45)	13 (0.64)	
Liver disorders			
No	53,151 (98.43)	1,990 (98.71)	0.32
Yes	847 (1.57)	26 (1.29)	
Gestational diabetes			
No	45,782 (84.78)	1,732 (85.91)	0.17
Yes	8,216 (15.22)	284 (14.09)	
Multiple births			
No	52,682 (97.56)	1,936 (96.03)	<0.01
Yes	1,316 (2.44)	80 (3.97)	
Macrosomia			
No	51,016 (94.48)	1,813 (89.93)	<0.01
Yes	2,982 (5.52)	203 (10.07)	
Maternal outcomes			
Total	53,998 (100.0)	2,016 (100.0)	–
Critical condition	129 (0.24)	175 (8.68)	
Death	1 (0.00)	1 (0.05)	
Perinatal outcomes			
Total	55,319 (100.0)	2,100 (100.0)	–
Alive	55,084 (99.58)	2,087 (99.38)	
Neonatal asphyxia	1,314 (2.39)	87 (4.17)	
Stillbirth	235 (0.42)	13 (0.62)	

### Risk factors for PPH

Univariate analyses revealed significant differences between the PPH and non-PPH groups in terms of education level, number of prenatal checkups, mode of delivery, history of cesarean section, multiple births, hypertensive disorders of pregnancy, and macrosomia (all *P* < 0.05). After adjusting for potential risk factors, multiple births ([OR] 3.10; 95% [CI] 2.42–3.98; *P* < 0.01), vaginal delivery (OR 3.00; 95% CI 2.62–3.42; *P* < 0.01), macrosomia (OR 2.30; 95% CI 1.97–2.68; *P* < 0.01), hypertensive disorders of pregnancy (OR 1.79; 95% CI 1.50–2.13; *P* < 0.01), and lower education level (OR 1.20; 95% CI 1.07–1.36; *P* < 0.01) emerged as independent risk factors for PPH (Table 2).

**Table 2:** Risk factors for PPH.

Characteristics	Unadjusted model^a^		Adjusted model^b^	
	OR (95% CI)	*P*	OR (95% CI)	*P*
Education level				
Higher education	1		1	
High school	1.14 (1.01–1.27)	0.03	1.21 (1.08–1.36)	<0.01
Junior high school or below	1.15 (1.02–1.29)	0.02	1.20 (1.07–1.36)	<0.01
Number of prenatal checkups				
<5	1		1	
≥5	0.67 (0.51–0.88)	<0.01	0.80 (0.60–1.05)	0.11
Mode of delivery				
Cesarean section	1		1	
Vaginal delivery	2.88 (2.59–3.22)	<0.01	3.00 (2.62–3.42)	<0.01
History of cesarean section				
No	1		1	
Yes	0.30 (0.26–0.37)	<0.01	0.65 (0.54–0.80)	<0.01
Hypertensive disorders of pregnancy				
No	1		1	
Yes	1.47 (1.24–1.74)	<0.01	1.79 (1.50–2.13)	<0.01
Multiple births				
No	1		1	
Yes	1.65 (1.31–2.08)	<0.01	3.10 (2.42–3.98)	<0.01
Macrosomia				
No	1		1	
Yes	1.92 (1.65–2.23)	<0.01	2.30 (1.97–2.68)	<0.01

Notes:

^a^Univariate analysis via logistic regression.

^b^Multivariate analysis after adjusting for education level, number of prenatal checkups, mode of delivery, history of cesarean section, hypertensive disorders of pregnancy, multiple births, and macrosomia.

Stratified analysis of the mode of delivery indicated that macrosomia (OR 2.87; 95% CI 2.42–3.40; *P* < 0.01) increased the risk of PPH in women undergoing vaginal delivery. For women undergoing cesarean section, > 4 prenatal checkups (OR 0.51; 95% CI 0.28–0.92; *P *= 0.03) was associated with a significantly lower risk of PPH (Table 3).

**Table 3:** Risk factors for PPH according to mode of delivery.

Characteristics	Total population^a^		Vaginal delivery^b^		Cesarean section^b^	
	OR (95% CI)	*P*	OR (95% CI)	*P*	OR (95% CI)	*P*
Education level						
Higher education	1		1		1	
High school	1.21 (1.08–1.36)	<0.01	1.16 (1.01–1.32)	0.03	1.44 (1.13–1.83)	<0.01
Junior high school or below	1.20 (1.07–1.36)	<0.01	1.14 (0.99–1.30)	0.07	1.49 (1.16–1.92)	<0.01
Number of prenatal checkups						
<5	1		1		1	
≥5	0.80 (0.60–1.05)	0.11	0.88 (0.64–1.19)	0.88	0.51 (0.28–0.92)	0.03
Mode of delivery						–
Cesarean section	1					
Vaginal delivery	3.00 (2.62–3.42)	<0.01				
History of cesarean section						
No	1		1		1	
Yes	0.65 (0.54–0.80)	<0.01	1.03 (0.68–1.56)	0.90	0.55 (0.44–0.68)	<0.01
Hypertensive disorders of pregnancy						
No	1		1		1	
Yes	1.79 (1.50–2.13)	<0.01	1.60 (1.26–2.02)	<0.01	2.00 (1.54–2.60)	<0.01
Multiple births						
No	1		1		1	
Yes	1.65 (1.31–2.08)	<0.01	3.85 (2.29–6.50)	<0.01	2.58 (1.94–3.43)	<0.01
Macrosomia						
No	1		1		1	
Yes	2.30 (1.97-2.68)	<0.01	2.87 (2.42-3.40)	<0.01	1.09 (0.75-1.58)	0.65

Notes:

^a^Multivariate analysis after adjusting for education level, number of prenatal checkups, mode of delivery, history of cesarean section, hypertensive disorders of pregnancy, multiple births, and macrosomia.

^b^Multivariate analysis after adjusting for education level, number of prenatal checkups, history of cesarean section, hypertensive disorders of pregnancy, multiple births, and macrosomia.

## Discussion

In this study, the incidence of PPH in Zhejiang Province was 3.60%. Multiple births, vaginal delivery, macrosomia, hypertensive disorders of pregnancy, and lower education level were identified as independent risk factors for PPH. These risk factors varied in their impact on PPH among different methods of delivery and fetal numbers.

The incidence of PPH exhibits notable regional variations, ranging from 3.2%–6.4% [17–19] in developed countries to 9%–25% in developing countries [20,21]. With an incidence of 3.60%, Zhejiang Province’s rates are significantly lower than those in developing countries and align with rates in developed countries. The PPH mortality rate in this study was remarkably low at 1.7 per 100,000 live births, contrasting with the higher rate of 50 per 102,525 live births reported in Northeast India, a region with a similar demographic profile. This rate is comparable to that of France (1 per 100,000 live births) [22,23]. A systematic prevalence review across 26 studies found that the median maternal PPH mortality rate in low- and middle-income countries was 95 per 100,000 live births, significantly exceeding the 5 per 100,000 seen in high-income countries [24]. Evidence suggests that PPH incidence and mortality decrease substantially with improvements in economic and healthcare levels [25,26]. The economic and obstetrical care levels in Zhejiang Province are advanced. In 2020, the per capita gross domestic product in Zhejiang Province exceeded 105,000 yuan, surpassing the global average of 11,033 dollars. The per capita disposable income was 52,397 yuan; the ratio of health personnel to beds was 1.52, one of the highest China [27].

Multiple births, hypertensive disorders of pregnancy, and macrosomia have been confirmed as independent risk factors for PPH, in alignment with existing literature [28,29]. However, vaginal delivery was identified as a significant risk factor for PPH, which conflicted with previous reports [30–32], although it matched the results of a large cohort study that enrolled 18,435 pregnant women [33]. Discrepancies in PPH definitions and methods of estimating blood loss may contribute to the varying incidence rates between vaginal and cesarean deliveries. However, A retrospective analysis of 1,295 maternal cases indicated that the incidence of PPH was higher in women who delivered vaginally than in those who delivered by cesarean section regardless of whether the blood loss cutoff of 500 mL or 1,000 mL was chosen [34], potentially due to clinical tendencies to overestimate blood loss during cesarean sections and underestimate it during vaginal deliveries. Technological advances in cesarean delivery have gradually reduced PPH incidence in this mode of delivery. Additionally, for pregnant women at high risk of PPH, such as those with a scarred uterus, arterial embolization techniques are frequently employed, significantly mitigating PPH risk [35]. Weak uterine contractions and placenta-related complications were prevalent in women with PPH (S1 Table), particularly during vaginal delivery, correlating with higher PPH rates [36].

Additionally, we investigated differences in PPH risk factors based on the mode of delivery; macrosomia was associated with a high risk of PPH in women undergoing vaginal delivery, whereas its correlation with PPH in cesarean sections was less pronounced. These findings support previous research indicating that macrosomia elevates the likelihood of PPH by intensifying intrapartum high-risk factors. There is evidence linking macrosomia with dystocia, protracted labor-induced uterine atony, lacerations of the soft birth canal, and extensive placental separation [37], all identified as risk factors for PPH in vaginal deliveries, culminating in a higher incidence. To mitigate these risks, prenatal checkups should intensively monitor fetal growth and development, with vigilant oversight of vaginal deliveries. Should signs of uterine atony emerge during labor, prompt medication to induce uterine contractions is essential. Enhanced midwifery techniques are also crucial in diminishing the risk of PPH.

We also observed that among women who had cesarean sections, those with five or more prenatal checkups experienced a reduced risk of PPH. This observation is supported by a retrospective analysis of 132 women [38]. Frequent prenatal checkups facilitate the identification of high-risk pregnancies, helping to manage pregnancy complications, control disease progression, and provide timely health education and guidance. Thus, they diminish PPH risk in cesarean deliveries [39]. Simultaneously, a cross-sectional study of 749 pregnant women demonstrated a positive correlation between the number of prenatal checkups and family economic income. Women who underwent more than four prenatal checkups displayed greater economic resources compared to those who had four or fewer. Therefore, they are better positioned to access new health care knowledge and higher-quality health services, thereby reducing the risk of PPH [40]. Initiatives such as creating medical records for pregnant women, devising personalized plans with follow-up reminders, and offering tiered subsidies for prenatal checkups based on economic status can enhance adherence to regular prenatal visits. Such measures are crucial in lowering the incidence of PPH, particularly in cesarean deliveries. However, the relationship between the frequency of prenatal checkups and the onset of PPH has been minimally investigated.

This study also identified an association between educational level and PPH incidence. Lower educational attainment was linked to higher PPH rates, possibly due to younger age and economic disadvantages hindering access to maternal and child health information. This lack of awareness about PPH precludes necessary interventions, a theory supported by a recent survey of 318 Chinese women [41]. Enhancing educational programs for expectant mothers and bolstering both digital and physical platforms for knowledge dissemination can substantially lower PPH rates; further studies are required to substantiate this link.

Evidence suggests that pregnancy comorbidities and multiple births are significant risk factors for PPH [29], potentially leading to intrauterine distress and adverse neonatal outcomes such as stillbirth or asphyxia [2], as also demonstrated in other extensive research [3]. Therefore, reducing PPH incidence is vital for improving maternal and perinatal health and crucial in reducing mortality.

In this study, the two maternal fatalities occurred in district hospitals; one was due to PPH following complications from gestational hypertension and a lack of prenatal care. During a forceps-assisted vaginal delivery, the woman developed amniotic fluid embolism, leading to disseminated intravascular coagulation (DIC), massive hemorrhage, and eventual death after failed interventions. The other death resulted from a subarachnoid hemorrhage complicated by brain herniation. Factors contributing to these outcomes included inadequate prenatal assessment, insufficient midwifery skills at primary care facilities, and delayed referrals. Enhancing facility capabilities to monitor and manage high-risk pregnancies, alongside strengthening midwifery training and labor management, is imperative for reducing maternal mortality risks.

The primary strength of this study was its extensive sample size, encompassing hospitals at provincial, municipal, and county levels, including 11 tertiary and 7 secondary facilities. The study population was representative, and the incorporation of multiple research centers yielded a diverse dataset that enhanced the robustness and generalizability of the findings. The large number of participants also increased the statistical power, facilitating more detailed and reliable conclusions. However, the study had several limitations. First, the data were sourced from the Zhejiang Province MNMSS, and case information might have been underreported. Critical maternal characteristics related to PPH, such as body weight, body mass index, and assisted reproductive technology treatment, were not recorded, potentially introducing confounding bias. Secondly, inconsistencies in PPH measurement methods may have led to errors in PPH estimation. Despite this, the PPH variables were primarily based on clinical diagnoses in medical institutions, aligned with current clinical protocols. Thirdly, the cross-sectional nature of the study was time-sensitive and only reflected the situation in Zhejiang Province at the time of investigation. Finally, the study was geographically limited to Zhejiang Province, significantly restricting the applicability of its findings to other regions.

## Conclusion

The incidence of PPH in Zhejiang Province was 3.60%. The study identified multiple births, vaginal delivery, macrosomia, hypertensive disorders of pregnancy, and low educational levels as independent risk factors for PPH. These identified risk factors may be instrumental in efforts to reduce PPH incidence. Enhanced antenatal care is crucial for timely identification of PPH risk factors, and improved labor management and midwifery skills are essential in mitigating the risk during vaginal deliveries. Particularly following the relaxation of the maternity policy, these measures are vital for increasing natural birth rates, managing cesarean section rates, and improving maternal and infant health outcomes.

## Supporting information

S1 Table Factors associated with PPH. (DOC)

S1 Dataset Minimal data set. (XLSX)
