# Supplementary material for: Postpartum hemorrhage incidence and risk factors: Evidence from a multicenter study in Zhejiang Province, China
Source: PLoS One. 2025 May 29;20(5):e0323190. doi: 10.1371/journal.pone.0323190 (PMC12121736; doi:10.1371/journal.pone.0323190)
Supplement: S1 Table — (DOC) [file pone.0323190.s001.doc]

S1 Table Factors associated with postpartum hemorrhage

| Factors | Number (*n*=2 016) | Ratio (%) |
| --- | --- | --- |
| Uterine atony | 1 820 | 90.28 |
| Placenta associated complications | 346 | 17.16 |
| Placenta previa | 147 | 7.29 |
| Retained placenta | 138 | 6.85 |
| Placental abruption | 61 | 3.02 |
| Laceration of soft birth canal | 267 | 13.24 |
| Coagulation disorders | 168 | 8.33 |
